# The discovery of novel noncoding RNAs in 50 bacterial genomes

**DOI:** 10.1093/nar/gkae248

**Published:** 2024-04-22

**Authors:** Aya Narunsky, Gadareth A Higgs, Blake M Torres, Diane Yu, Gabriel Belem de Andrade, Kumari Kavita, Ronald R Breaker

**Affiliations:** Department of Molecular, Cellular and Developmental Biology, Yale University, New Haven, CT 06511, USA; Department of Molecular, Cellular and Developmental Biology, Yale University, New Haven, CT 06511, USA; Department of Molecular, Cellular and Developmental Biology, Yale University, New Haven, CT 06511, USA; Department of Molecular, Cellular and Developmental Biology, Yale University, New Haven, CT 06511, USA; Department of Molecular, Cellular and Developmental Biology, Yale University, New Haven, CT 06511, USA; Department of Molecular, Cellular and Developmental Biology, Yale University, New Haven, CT 06511, USA; Department of Molecular, Cellular and Developmental Biology, Yale University, New Haven, CT 06511, USA; Department of Molecular Biophysics and Biochemistry, Yale University, New Haven, CT 06511, USA; Howard Hughes Medical Institute, Yale University, New Haven, CT 06511, USA

## Abstract

Structured noncoding RNAs (ncRNAs) contribute to many important cellular processes involving chemical catalysis, molecular recognition and gene regulation. Few ncRNA classes are broadly distributed among organisms from all three domains of life, but the list of rarer classes that exhibit surprisingly diverse functions is growing. We previously developed a computational pipeline that enables the near-comprehensive identification of structured ncRNAs expressed from individual bacterial genomes. The regions between protein coding genes are first sorted based on length and the fraction of guanosine and cytidine nucleotides. Long, GC-rich intergenic regions are then examined for sequence and structural similarity to other bacterial genomes. Herein, we describe the implementation of this pipeline on 50 bacterial genomes from varied phyla. More than 4700 candidate intergenic regions with the desired characteristics were identified, which yielded 44 novel riboswitch candidates and numerous other putative ncRNA motifs. Although experimental validation studies have yet to be conducted, this rate of riboswitch candidate discovery is consistent with predictions that many hundreds of novel riboswitch classes remain to be discovered among the bacterial species whose genomes have already been sequenced. Thus, many thousands of additional novel ncRNA classes likely remain to be discovered in the bacterial domain of life.

## Graphical Abstract

**Graphical Abstract:**
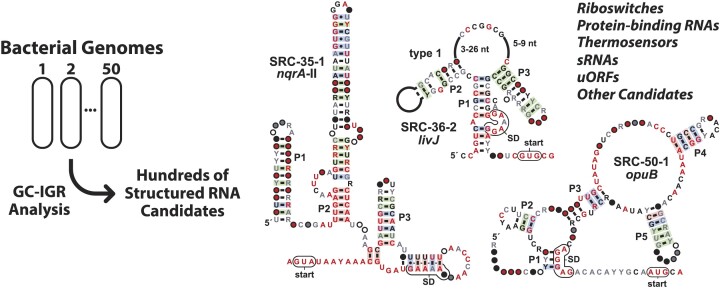

## Introduction

Many types of noncoding RNAs (ncRNAs) reside in bacterial species where they perform numerous tasks ranging from key roles in protein production (e.g. tRNAs and rRNAs) (1,2) to the regulation of gene expression (e.g. sRNAs and riboswitches) (3–6). In addition to these ancient and widespread classes of ncRNAs involved in translation and gene regulation, numerous other ncRNA classes are known to be broadly distributed and carry out other important functions. For example, some bacterial RNAs fold to form ribozymes that catalyze RNA processing (e.g. RNase P) through phosphoester hydrolysis (7) and numerous self-cleaving ribozymes that perform internal phosphoester transfer reactions (8). Other ncRNAs derived from various CRISPR systems (9,10) serve as guides for protein enzymes that target nucleic acids for modification or degradation. These ncRNAs and many other known classes likely represent only a sampling of the true diversity of biochemically active RNAs that exist in the bacterial domain of life.

A major challenge for discovery of novel bacterial ncRNA classes is that current biochemical, genetic, or bioinformatic methods are limited in their ability to identify candidates efficiently and comprehensively. Most biochemical and genetic methods, such as RNA structural probing (11) or RNA transcriptomics (12), are generally limited to examining the RNAs produced in a single, culturable organism per experiment. Thus, other strategies are needed to effectively uncover additional ncRNA candidates more efficiently.

Computer-aided search methods can be used to avoid experimental bottlenecks by rapidly searching large genomic DNA datasets. These bioinformatics approaches mostly exploit comparative sequence analysis programs that benefit from searching many diverse genomes carrying highly conserved representatives of ncRNA targets (e.g. (13,14)). Bioinformatics-based ncRNA discovery efforts are progressing, but it is likely that the more common classes will be uncovered sooner because greater numbers of representatives provide more opportunities for comparative sequence analysis programs to encounter hits. Unfortunately, it is expected to become progressively more difficult to identify additional, rare ncRNA classes because fewer representatives of undiscovered classes will be present in genomic sequence databases.

To improve the prospects of identifying strong candidates for novel structured ncRNAs, a strategy was developed (15–17) to focus comparative sequence analysis searches on regions of bacterial genomes that are most likely to serve as templates for the transcription of ncRNAs. A version of this strategy, called the ‘GC-IGR search’ approach (18), was utilized by our laboratory to more comprehensively identify structured noncoding RNAs encoded by the genome of each bacterial species. Previously (17–19), we examined the genomes of 31 bacterial species and identified numerous candidate ncRNAs including 18 candidate riboswitch classes. Riboswitches are ncRNA domains commonly residing in the 5′-untranslated region (5′-UTR) of certain mRNAs where they bind target ligands and enhance or suppress the expression of associated genes by various mechanisms (20–23).

To date, three novel riboswitch classes from these 18 candidates have been experimentally validated and have been found to sense ligands relevant to enzyme cofactors: *S*-adenosylmethionine (SAM) (17), 4-amino-5-hydroxymethyl-2-methylpyrimidine pyrophosphate (HMP-PP) (24), and nicotinamide adenine dinucleotide (NAD^+^) (25). One of the 18 candidates also forms a complex structure and has been proven to regulate gene expression by another mechanism (26). Although there are 14 candidates from these initial GC-IGR searches remaining to be experimentally examined, we believe this approach remains a valuable way to uncover novel ncRNA motifs relevant to bacterial gene regulation.

The overall potential for discovering additional riboswitch classes is also likely to be enormous. It has been proposed (27) that the abundances of riboswitch classes follow a power law (28). By exploiting this relationship, we have predicted that many thousands of bacterial riboswitch classes remain to be discovered (20,22). To continue the search for riboswitch classes and other ncRNA motifs, we have applied the GC-IGR approach to 50 additional bacterial genomes. Our results reveal the existence of numerous novel structured ncRNA motifs, including 44 candidate riboswitch classes and other potential regulatory RNA candidates. This pace of discovery reinforces the hypothesis that the bacterial domain of life remains replete with undiscovered ncRNA structures.

## Materials and methods

### Choosing bacterial genomes for GC-IGR analysis

Genomes analyzed in this study were selected from complete genomes in the Reference Sequence (RefSeq) database release 76 (29). The guidelines for selecting the genomes focused on expanding the variety of phyla whose species have been subjected to the GC-IGR analysis. Genomes carrying <100 IGRs conforming to our analysis criteria (see below) were usually selected for full examination, which reduces the amount of manual intervention required for data generation and curation.

### The GC-IGR pipeline

The GC-IGR pipeline was implemented as previously described (18,19). Briefly, each IGR from a given genome was evaluated to establish its percent content of G and C nucleotides (%GC) and its length in nucleotides (IGR length). For bacterial species with relatively AT-rich genomes, IGRs that serve as transcription templates for structured ncRNAs tend to be, on average, higher in %GC (15–17) and IGR length (17) values than other IGRs. Thus, sequences representing ncRNAs tend to cluster towards the upper right quadrant of a plot of IGRs sorted based on these two characteristics.

To identify novel ncRNA representatives using this plot, a region encompassing most IGRs known to represent ncRNA sequences is identified, wherein this area is made large enough to also include an approximately equal number of ‘unknown IGRs’ with similar %GC and IGR length values but do not carry a known ncRNA or other genetic feature. BLASTX (30) was then applied to each of the unknown IGRs in this plot region to identify and remove IGRs that contain a known protein coding region. The remaining unknown IGRs were used by Infernal 1.1 (31) as queries to search RefSeq release 80 (RefSeq 80) (32) and a dataset of metagenomic DNA sequences (14) to find additional homologous representatives among other IGRs in the same species and in other species. Each IGR found to carry a representative of a known ncRNA class was classified as ‘Known RNA’ and removed from subsequent analysis steps. CMFinder (33) was used to develop predicted RNA secondary structure models from the sequence alignments of the remaining IGRs.

The sequence alignments and structure models were then queried again using Infernal 1.1 to generate larger sets of representatives for the IGRs, now termed ‘motifs’. This step is usually iterated, and the alignments and structures were refined by visual inspection and manual alteration to improve sequence alignments and structural models. We use JalView (34) to prepare initial editable sequence alignments, and then use Emacs with the RALEE plugin (35) to improve the alignments and secondary structure models. The consensus sequence and structural model for each motif is visually rendered using R2R (36) using its default parameters.

The BLISS server (Breaker Laboratory Intergenic Sequence Server) (13) is used to display the motifs and their surrounding genes, which are clues used to predict a function for each motif. Genes immediately adjacent to the motif are usually convincingly annotated and provide clues regarding the possible function of the RNA. In some instances, the identities of the adjacent genes are uncertain. For these, we use BLAST (37), HMMER (38) and HHPRED (39) to search for homologs. Based on these analyses, the predicted functions of the gene products are used as aids in predicting the possible function of the RNA motif.

### Categorizing IGRs and candidate RNA motifs

Following the nomenclature used in previous studies applying the GC-IGR pipeline (18,19), potential ncRNA motifs are classified into five main groups as follows:

- Unnamed: Insufficient evidence to classify.
- Low-ranking candidate (LRC): Usually fewer than five unique representatives and a poor consensus model.
- Medium-ranking candidate (MRC): Usually fewer than 20 unique representatives and/or a poor consensus model.
- High-ranking candidate (HRC): Many representatives and a good consensus model, but insufficient information regarding possible function.
- Named candidate: Could be rare, but usually has many representatives with a good consensus model and some evidence supporting a hypothesis for function.

Individual categories of ‘Named Candidates’ are listed and defined below.

*Strong riboswitch candidate (SRC)*. An SRC is a motif that usually is abundantly represented, exhibits evidence for complex secondary structure formation (abundant, conserved base-paired substructures), and resides in the 5′-UTR of an mRNA whose protein product is relevant to metabolic pathways, transporters, or other functions typical of riboswitch association (40). An expression platform might also be evident from the sequence alignment.

*Weak riboswitch candidate (WRC)*. A WRC has characteristics like an SRC but, comparatively, is rarer and has less evidence for forming a complex structure, an expression platform, or other characteristics common for riboswitches.

*Upstream open reading frame (uORF)*. A uORF candidate is a motif found upstream of a main ORF that exhibits conserved start and stop codons and has a pattern of sequence conservation usually consistent with a protein-coding region. Translation of the uORF likely regulates the expression of the main ORF (41).

*Protein binding candidate (PBC)*. A PBC is a motif located in the 5′-UTR of an mRNA typically coding for nucleic acid binding proteins. The protein likely regulates its own production by binding to its mRNA (42).

*Ribosomal leader candidate (RLC)*. An RLC is a type of PBC that is located in the 5′-UTR of an mRNA coding for a ribosomal protein. This is a common mechanism by which ribosomal proteins regulate their production (43).

*RNA thermometer candidates (RTC)*. An RTC is located in the 5′-UTR of a gene coding for proteins whose production is regulated by changes in temperature (44).

*Mobile/repeat sequence candidates (MRSC)*. An MRSC is predicted to be a selfish nucleic acid element. This prediction is based on the observation that the motif is present in multiple copies per genome and is often associated with genes coding for transposases, integrases, nucleases, or helicases (45).

*Small RNA (sRNA)*. An sRNA typically exhibits minimal secondary structure features, is not consistently located in the 5′-UTRs of mRNAs, and lacks consistent gene associations. Note that our computational search approach is not designed to comprehensively identify sRNAs and sORFs.

*Small open reading frame (sORF)*. An sORF exhibits conserved start and stop codons and a conservation pattern compatible with a coding region. Unlike a uORF, sORFs are not consistently located in a position to regulate the expression of a main ORF.

*Terminator stem candidate (TSC)*. A TSC forms a single, strong hairpin with robust evidence for covariation consistent with this stem. In addition, the hairpin is followed by a series of five or more U nucleotides (46,47).

## Results and discussion

### Genome and IGR selection and analysis

A total of 50 bacterial genomes were chosen for analysis to extend the number of bacterial phyla examined using the GC-IGR pipeline (18,19). In addition, genome choices were made to favor those having low %GC content and that yield ‘unknown IGRs’ with %GC content and length similar to known ncRNAs in numbers that are feasible for analysis. The genomic dataset analyzed includes species from 16 different phyla (Supplementary Table S1), expanding the total number of phyla analyzed by the GC-IGR pipeline to 19 (17–19). Overall, 4714 IGRs with lengths and %GC content like those of known ncRNAs were examined, of which 1274 were selected for in-depth analysis (Table 1).

**Table 1.** Summary of the motif candidates and their representatives uncovered in this study

Motif category	Total counts
Known ORFs	2948
Low-ranking candidates	607
Unnamed	427
Mobile/repeat sequence candidates	250
Medium-ranking candidates	110
Protein-binding candidates	107
Known RNAs	65
High-ranking candidates	55
Terminator stems	48
Weak riboswitch candidates	33
Ribosomal leader candidates	30
Strong riboswitch candidates	10
sRNA candidates	10
uORF candidates	8
sORF candidates	3
RNA thermometer candidates	2
Other named candidates	1
Total	4714

Each candidate IGR was examined for evidence of secondary and tertiary structure formation. For example, nucleotide positions that covary in a manner that retains base pairing is a strong indication that these indeed participate in secondary structure formation. Similarly, widespread conservation of nucleotide identities is consistent with the formation of extensive tertiary structures. IGRs exhibiting evidence of secondary and tertiary structure formation could represent novel ncRNA motifs, which are then sorted based on several factors used to support speculation on their putative functions. For example, a motif classified as a strong riboswitch candidate (SRC) usually has evidence of conserved nucleotide sequences and secondary structures, has features of a riboswitch expression platform, is consistently located in the predicted 5′-UTR of mRNAs, and is commonly associated with genes relevant to specific metabolic pathways and transporters (17–19).

As expected based on past GC-IGR analyses (17–19), we encountered genetic elements and structured ncRNA candidates that represent the full range of IGR and ncRNA categories described above. These results, which are summarized in the following sections, reveal that many bacteria carry novel functional motifs in noncoding regions of their genomes. Given the enormous diversity of bacterial species, the opportunity for discovering structured ncRNA classes using the GC-IGR approach or other discovery methods remains high.

### Strong riboswitch candidates

**Figure 1.**
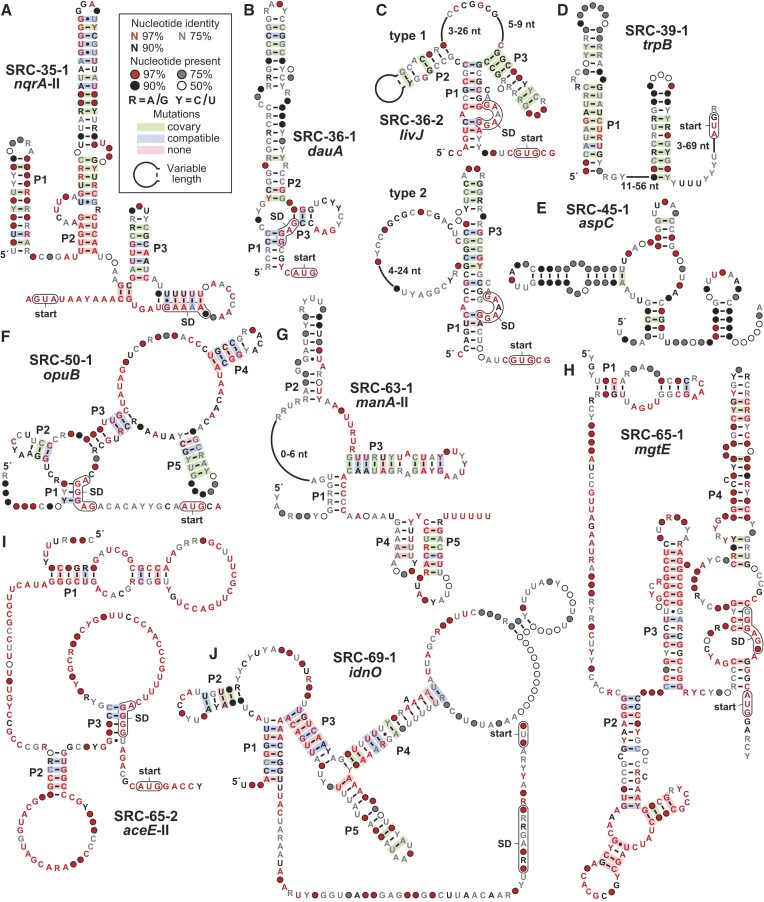
Consensus sequence and structural models of predicted strong riboswitch candidates. (**A**) *nqrA*-II motif. (**B**) *dauA* motif. (**C**) *livJ* motif. (**D**) *trpB* motif. (**E**) *aspC* motif. (**F**) *opuB* motif. (**G**) *manA*-II motif. (**H**) *mgtE* motif. (**I**) *aceE*-II motif. (**J**) *idnO* motif. Annotations for all motifs are as defined in (A). Shine-Dalgarno (SD) sequence regions are speculative. Sequence alignments for all motifs are provided as supplementary *sto* files.

We have organized novel riboswitch candidates uncovered in this study into strong (SRC) and weak (WRC) candidates. The distinction between the two is not precise, and only roughly reflects our level of confidence that a motif merits further investigation as a possible riboswitch class. These judgements are made by identifying and evaluating characteristics discernible by bioinformatics that are consistent with riboswitch function, including robust sequence conservation, strong evidence of forming complex structures, gene associations and orientations, and features consistent with expression platform function. Our analyses uncovered 11 previously unknown SRC motifs that exhibit most or all the characteristics indicative of riboswitch function. Representatives of one of these classes has already been reported by us (48) and by others (49) to function as riboswitches for guanidinium (called the guanidine-IV riboswitch class). The characteristics of the remaining ten candidates (Figure 1) are briefly described below.

Note that we have not tested most of the candidate riboswitch classes identified in the current study due to the myriad challenges in validating orphan riboswitches (50–52). For some candidates, we do not have sufficient clues to accurately identify the most likely riboswitch ligand. Even when a ligand becomes obvious for testing, often several RNA constructs need to be examined to ensure that an appropriate-sized (and functional) RNA construct is being evaluated. In some instances, the most likely riboswitch ligand to test is not commercially available, or is highly unstable. These and other complications make it impractical to test all candidates in a short timeframe and with limited resources.

The *nqrA*-II motif (SRC-35–1). A total of 36 unique (non-redundant) representatives of the *nqrA*-II motif (Figure 1A) were identified from five different genera of the *Pasteurellaceae* family. Representatives are consistently found upstream of the *nqrA* ORF whose protein product is annotated as a sodium-translocating NADH:quinone oxidoreductase (subunit A) (53). This protein is a component of the NQR complex, which catalyzes the reduction of ubiquinone-1 to ubiquinol and is coupled to a sodium transporter that pumps Na^+^ from the cytoplasm to the periplasm.

The consensus sequence and structural model of the *nqrA*-II motif includes four stem-loop structures (the paired substructures are called P1 through P4) that have strong support from sequence covariation. Specifically, when a mutation occurs in a nucleotide within a predicted stem, its partner often also mutates to preserve the base-paired structure. In addition, numerous conserved nucleotides are interspersed in or near the predicted stem-loop structures, which is typical of RNAs whose complex secondary and tertiary structures are preserved through evolution. An RNA structural analysis method called in-line probing (54,55) yields results that are consistent with the proposed secondary structure model (Supplementary Figure S1). Furthermore, *nqrA*-II motif representatives are located immediately upstream of the start codon for the *nqrA* ORF, which is consistent with a possible role for the motif in gene regulation.

Previously, we identified a different, rare motif also located upstream of *nqrA* ORFs in the genus *Marinomonas* (14) that was named the *nqrA* motif. Therefore, we have termed this newly identified motif as *nqrA*-II. Although there are no similarities between the two motifs, they might both function as riboswitches to sense the same ligand. Previously discovered Na^+^-sensing riboswitches are known to regulate genes relevant to Na^+^ ion transport and the establishment of Na^+^ gradients (56). Thus, *nqrA*-II motif RNAs might sense Na^+^ or another ligand that is relevant to cation gradients.

The *dauA* motif (SRC-36-1). A total of 57 unique representatives of the *dauA* motif (Figure 1B) have been identified from species of *Streptomyces*. The three-stem junction formed by these RNAs includes the putative Shine-Dalgarno (SD) sequence (57) located upstream of the start codon for the adjoining gene. This associated gene codes for a Sulfate Transport and Anti-Sigma factor antagonist (STAS) protein (58), suggesting the RNA might function as a genetic ‘OFF’ switch that responds to abundant sulfate or its surrogate. If this idea is correct, the proposed structure likely represents the ligand-bound conformation, which would block the SD and prevent ribosome binding to suppress gene expression.

Proteins carrying a STAS domain are also known to be involved in various other cellular functions (58,59). Therefore, the ligand for this riboswitch might not have a connection to sulfate, but rather is another ligand whose identity is not obvious to us based on current information. Furthermore, we cannot rule out the possibility that this or other candidates identified in this study are directly bound by protein factors to regulate RNA structure formation and gene expression.

The *livJ* motif (SRC-36–2). The *livJ* motif has 235 unique representatives and is mostly found in *Streptomyces* species. Representatives are located upstream of genes coding for LIVBP-like protein domains, which are present in the periplasmic binding domain of ABC transporters for all three branched chain amino acids (60). The representatives can be divided into two types that are grouped based on whether a P2 stem can easily be discerned (Figure 1C). The SD sequence is predicted to reside in an internal bulge of P1, which could be well suited to undergo sequestration based on the ligand binding status of the RNA. We speculate that this motif might function as a riboswitch that senses leucine, isoleucine, valine, or another surrogate for these branched chain amino acids to reduce gene expression upon ligand binding.

The *trpB* motif (SRC-39-1). More than 900 distinct representatives of the *trpB* motif are present in *Firmicutes* and *Fusobacteria*. The motif (Figure 1D) is almost always located in the 5′-UTR of the associated gene, although the identity of this gene varies. The motif is occasionally located upstream of *trpB*, which codes for a component of tryptophan synthase (61). However, the most commonly associated gene codes for an uncharacterized ABC transporter with strong homology to amino acid transporters. Thus, we speculate that the *trpB* motif could represent a riboswitch class that senses a ligand relevant to amino acids metabolism.

Strong evidence for covariation supports a model where the motif can form at least two hairpins. The second of these substructures has the characteristics of an intrinsic terminator, including a strong base-paired stem followed by a run of U nucleotides (46,47). Riboswitches often exploit intrinsic terminator stems to regulate gene expression (20–23), and *trpB* motif RNAs appear to carry an anti-terminator region in the right shoulder of P1. However, we cannot rule out the possibility that the motif binds a protein factor or functions as a classic attenuator system like those known to regulate genes relevant to tryptophan metabolism (62).

The *aspC* motif (SRC-45-1). With over 1000 representatives, the *aspC* motif (Figure 1E) is the most widespread of the 11 strong riboswitch candidates and one of the most wide-spread motifs uncovered in this study. However, only 11 representatives are found in defined species whereas all others were identified from environmental DNA sequence datasets. When its gene association can be determined, the motif is located immediately upstream of the start codon for a gene coding for a putative amino acid aminotransferase (63). Thus, the motif might regulate the expression of the adjoining gene by sensing the abundance of a specific amino acid. Although the motif appears to form a three-stem junction and another putative stem, there are very few highly conserved nucleotides, which somewhat dampens our enthusiasm for this motif as a riboswitch candidate.

The *opuB* motif (SRC-50-1). The *opuB* motif has 205 distinct representatives mostly from species of *Enterobacteriaceae*. The consensus sequence and secondary structure model (Figure 1F) includes five base-paired substructures and stretches of highly conserved nucleotides, which strongly indicate the RNA forms a complex architecture. In addition, the P1 stem is formed in part using nucleotides of the SD sequence for the associated ORF, suggesting that ligand binding will suppress ribosome engagement and gene expression. Preliminary genetic analysis indicates that a representative *opuB* motif RNA functions as a riboswitch that activates gene expression upon binding ligand, and that the ligand is present in cells grown under rich media conditions (Supplementary Figure S2).

The most common downstream-adjacent gene, *opuB*, is annotated as coding for a proline/glycine ABC-transporter, whereas other related genes are known to be relevant to the transport of compatible solutes such as glycine, betaine, and choline (64). Notably, choline has previously been reported to affect the expression of an *opuB* gene (65). Strong genetic and biochemical evidence indicates that the *opuB* motif indeed functions as a riboswitch, and its natural ligand appears to be relevant to shikimate biosynthesis and aromatic amino acid utilization (Breaker Laboratory, unpublished findings).

The *manA*-II motif (SRC-63-1). The *manA*-II motif is found in 85 examples in *Leptotrichia* species, where it is found upstream of the following two genes. One is annotated as *manA*, which codes for mannose-6-phosphate isomerase (66) and the other is similar to *pflA*, which is a glycyl-radical enzyme activating protein (67). The motif is composed of a 3-stem junction followed by two additional hairpins, wherein the last appears to be a transcription terminator (Figure 1G). Thus, ligand binding might regulate the formation of the terminator stem to control transcription of the adjacent gene.

The *mgtE* motif (SRC-65-1). A total of 32 unique representatives of the *mgtE* motif were identified exclusively in the genus *Thioalkalivibrio*. MgtE proteins are known to be magnesium transporters, but metal ion transporters are often misannotated and so the associated gene might code for a transporter of a different ligand. The consensus sequence and structure model for the motif includes the SD and AUG start codon for the associated ORF at the bottom of a large and imperfect P4 stem (Figure 1H). The predicted secondary structure model is supported by evidence for nucleotide covariation that maintains base pairing. Although the model also includes numerous highly conserved nucleotides, the narrow distribution of the representatives might cause an overestimate of the positions whose nucleotide identity is critical for function of the RNA motif.

The *aceE*-II motif (SRC-65–2). The *aceE*-II motif has 25 unique representatives, all from the *Thioalkalivibrio* genus, and this might lead to its unusually high number of conserved nucleotides (Figure 1I). The motif is located exclusively upstream of the *aceE* gene, which codes for pyruvate dehydrogenase subunit E1. The enzyme, which is one of the components of the pyruvate dehydrogenase complex (68), converts pyruvate into acetyl-CoA and CO_2_. In species that carry the RNA motif, the *aceE* gene is the first in a large operon that includes genes for other components of the pyruvate dehydrogenase complex, including *aceF* (dihydrolipoamide acetyltransferase) and *lpdA* (dihydrolipoamide dehydrogenase). Thus, the ligand for this riboswitch candidate could be pyruvate or another molecule that is indicative of the potential for or need to regulate the production of this key process for energy and carbon utilization. Another riboswitch candidate, called the *aceE* motif, has been previously reported (14), and it is possible that the two motifs sense the same ligand.

The *idnO* motif (SRC-69–1). The *idnO* motif has 265 unique representatives, mostly from *Streptococcus* species but also from *Erysipelotrichaceae* genera. The motif occupies the entire IGR between two genes: *manX* (phosphotransferase system sugar transporter) and *idnO* (gluconate 5-dehydrogenase). Therefore, the RNA motif appears to be relevant to sugar metabolism. The motif is quite large and is interspersed with stem structures that are partly supported by evidence of nucleotide covariation (Figure 1J).

### Weak riboswitch candidates

**Figure 2.**
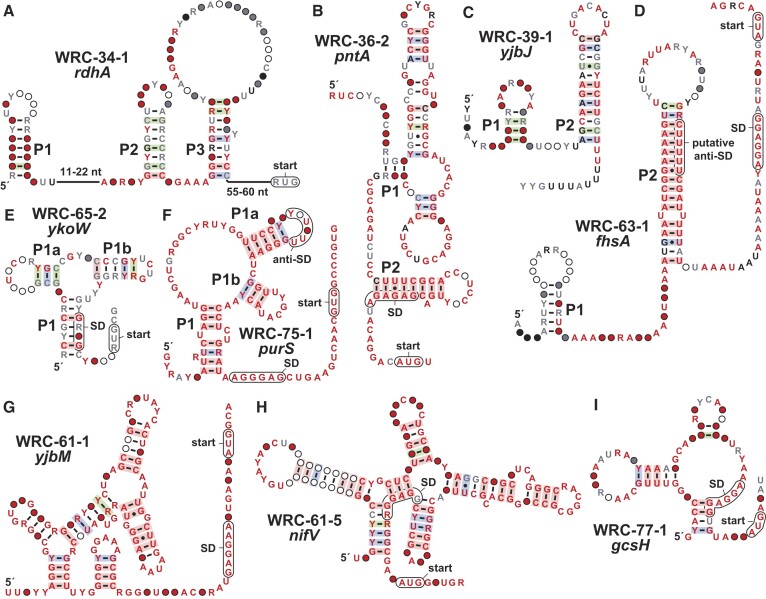
Consensus sequence and structural models of several weak riboswitch candidates. (**A**) *rdhA* motif. (**B**) *pntA* motif. (**C**) *yjbJ* motif. (**D**) *fhsA* motif. (**E**) *ykoW* motif. (**F**) *purS* motif. (**G**) *yjbM* motif. (**H**) *nifV* motif. (**I**) *gcsH* motif. Annotations for all motifs are as defined in Figure 1A. Shine-Dalgarno (SD) sequence regions are speculative.

Our analyses identified 33 previously unknown WRC motifs. The characteristics of nine of these candidates (Figure 2) are briefly described below, and the remainder are listed in the Supplementary Data (Supplementary Figure S3 and Supplementary Figure S4).

The *rdhA* motif (WRC-34–1). The *rdhA* motif (Figure 2A) has 18 unique representatives, mostly from *Dehalococcoides* species and others from environmental DNA sequence datasets. When gene association data is available, the motif is located immediately upstream of a gene annotated as coding for a reductive dehalogenase (69). This protein detoxifies chlorinated aromatic and aliphatic compounds. A previous study showed that the transcription of this gene in *Dehalococcoides mccartyi* is regulated by 2,3-dichlorodibenzo-*p*-dioxin (DCDD) (70).

The *pntA* motif (WRC-36–2). The *pntA* motif consists of only 11 unique representatives and is found exclusively in the *Streptomyces* genus. The motif is located upstream of the *pntA* gene which codes for NAD(P) transhydrogenase (71). Thus, the RNA structure might regulate the expression of the associated gene by sensing a form of nicotinamide adenine dinucleotide. The predicted secondary structure (Figure 2B) includes a P2 stem that includes nucleotides of the SD sequence for the associated ORF, suggesting that the presence of a ligand might stabilize P2 and prevent ribosome binding to suppress gene expression.

The *YjbJ* motif (WRC-39-1). The *YjbJ* motif has 40 unique representatives, all from the *Streptococcus* genus. The motif is located about 30 nucleotides upstream of the SD and AUG start codon of the associated ORF. The *yjbJ* gene is predicted to encode a lytic transglycosylase, that catalyzes cleavage of the peptidoglycan structures of the cell wall (72), but has additional roles in diverse cellular functions ranging from cell-wall synthesis, antibiotic resistance, and sporulation (73–75). The protein structure resembles the *csbD* family of bacterial general stress response proteins, thus previous studies suggested that *yjbJ* is also involved in stress responses (72).

Evidence of covariation supports our prediction (Figure 2C) that the motif forms two hairpins, the latter of which is preceded by a stretch of U nucleotides, consistent with the characteristics of an intrinsic terminator (46,47). Considering the diversity of cellular functions in which the *yjbJ* gene product is involved, the riboswitch candidate might sense a signaling molecule. However, the expression of the *yjbJ* homolog *csbD* is often mediated by the binding of a sigma factor (76). This serves as a reminder that this and other motifs might be involved in the binding of a protein factor.

The *fhsA* motif (WRC-63-1). The *fhsA* motif has 52 distinct representatives. Seven representatives are from *Leptotrichia* species and the rest are from environmental DNA sequence datasets. The *fhsA* gene codes for formate-tetrahydrofolate ligase. This enzyme uses ATP to catalyze the formylation of tetrahydrofolate to yield 10-formyltetrahydrofolate, which is a major component of the one-carbon pool (77). The predicted structure of the motif (Figure 2D) includes a P2 hairpin with characteristics of an intrinsic terminator. The stem-loop of the terminator could potentially form a pseudoknot with the preceding hairpin, which, in turn, could prevent the formation of the terminator and allow the RNA polymerase to transcribe through the downstream ORF. We speculate that tetrahydrofolate or one of its one-carbon derivatives might participate in regulation via this RNA motif.

The *ykoW* motif (WRC-65-2). Only 11 distinct representatives of the *ykoW* have been found, exclusively from the *Thioalkalivibrio* genus. The motif is located upstream of a gene for an uncharacterized protein which contains an EAL domain commonly associated with c-di-GMP hydrolysis (78). Despite the small number of unique representatives and their narrow phylogenetic distribution, there is sufficient diversity among these RNAs to observe evidence of covariation supporting the proposed three-stem junction (Figure 2E). P1 overlaps the SD sequence, and thus the motif may bind its target molecule to alter ribosome engagement with the mRNA as a mechanism of gene control.

The *purS* motif (WRC-75–1). With only six representatives exclusively from the *Corynebacterium* genus, the current consensus sequence for the *purS* motif is over-representing the highly conserved nucleotide positions (Figure 2F). Regardless, the RNA is proposed to adopt a complex structure wherein one of the loops could form an anti-SD sequence to block the ribosome from binding and translating the downstream ORF. The motif is located upstream of an operon that codes for phosphoribosylformylglycinamidine (FGAM) synthase, which catalyzes a step in the de novo purine biosynthesis pathway (79). The enzyme uses ATP to convert formylglycinamideribonucleotide (FGAR) and glutamine to FGAM and glutamate, by transferring an ammonia molecule from the glutamine to FGAR. Thus, candidate ligands could include purines or their biosynthetic intermediates.

The *yjbM* motif (WRC-61-1). There are only three representatives of the *yjbM* motif, found exclusively in the *Geobacter* genus. In the vast majority of cases with so few representatives, the candidate motif remains ‘unnamed’. However, its GC-rich character, complex structural model (Figure 2G), proximity to the SD and AUG sequences, and association with the *yjbM* gene induced us to classify the motif as a WRC. The *yjbM* gene codes for a (p)ppGpp synthetase (80), which might serve as the ligand for this riboswitch candidate.

The *nifV* motif (WRC-61–5). The *nifV* motif has four unique representatives that are all found in the *Geobacter* genus. It is located upstream of the *nifV* gene, which codes for homocitrate synthase (81). Homocitrate is an essential component of FeMo cofactor, located in the catalytic center of nitrogenases. The predicted intricate structure of the RNA (Figure 2H) is well supported by covariation despite the small number of representatives. The SD of the associated ORF is an integral part of the proposed structure, and thus ligand-mediate structural modulation could regulate ribosome engagement with the mRNA.

The *gcsH* motif (WRC-77–1). With only six representatives from two *Desulfobacteraceae* genera, the *gcsH* motif is another example of a rare, yet compelling motif. The motif (Figure 2I) is located upstream of *gcsH*, whose gene product is a part of the four-protein glycine cleavage system that decarboxylates glycine and plays a major role in glycine metabolism (82). Glycine riboswitches were previously reported that regulate genes relevant to glycine cleavage systems (83,84), however this motif is distinct from riboswitches known to regulate these genes.

### Other candidate structured nucleic acid motifs

**Figure 3.**
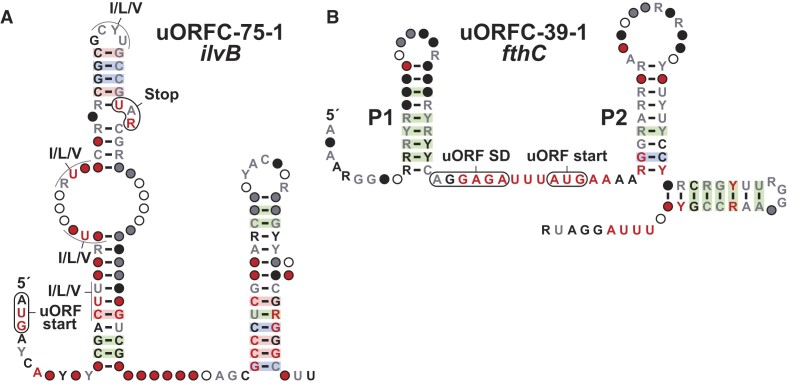
Consensus sequence and structural models of selected uORF candidates. (**A**) *ilvB* uORFC. (**B**) *fthC* uORFC. Annotations for all motifs are as defined in Figure 1A.

Although the initial classification of a motif as a *cis-*regulatory element may be correct, it is not trivial to predict the exact mechanism by which it controls gene expression. For example, the *ilvB*-II motif (uORFC-75-1) identified in the current study (Figure 3A) was originally considered a riboswitch candidate, potentially sensing ppGpp. However, further analysis revealed that it regulates gene expression by functioning as a uORF (85). Another example is the *pyrG* motif as described in the Supplementary Information (Supplementary Figure S5).

Predicting the function of a motif is especially challenging for rare motifs, which typically provide fewer clues regarding their functions. Thus, classifications of rare motifs should be considered highly speculative. In the sections below, we provide a few examples of candidate RNA or DNA motifs that appear to have functions that are distinct from riboswitch mechanisms.

*uORF candidates*. There are eight examples of uORF candidate in our dataset. We classify a motif as a uORF when its location is consistent with gene-control function, and the downstream gene identity is consistent. The motif will include conserved and in-frame start and stop codons, and often we can identify an associated SD sequence upstream of the uORF start codon.

The *ilvB* motif (uORFC-75-1). The *ilvB* motif has 103 unique representatives, all found in the *Corynebacterium* genus. The associated ORF is always the *ilvB* gene, coding for the large subunit of acetolactate synthase, which participates in the isoleucine, leucine and valine biosynthesis pathways. In firmicutes, this gene is sometimes regulated by a ppGpp riboswitch (80), which led us to consider this motif as a riboswitch candidate. However, further investigation of the motif revealed a conserved start codon, always in-frame with a stop codon. The motif includes two stems, termed P1 and P2 (Figure 3A). P2 has the characteristics of an intrinsic terminator, whereas P1 contains a complementary antiterminator sequence. The uORF is enriched in codons coding for isoleucine, leucine and valine amino acids, which are expected to cause ribosome stalling when levels of these amino acids are low. Translational stalling is then predicted to favor formation of the antiterminator stem, and transcription of the full mRNA (85).

The *fthC* motif (uORFC-39-1). The *fthC* motif has 69 unique representatives exclusively from the *Streptococcus* genus. The motif is formed by three hairpins (Figure 3B), where the third has characteristics of an intrinsic terminator stem. Stems P1 and P2 are separated by a stretch of conserved nucleotides that appear to encompass a purine rich SD-like region as well as a putative AUG start codon for a uORF. The P2 often carries a stop codon that resides in-frame with the proposed uORF start codon. The downstream main ORF codes for 5-formyltetrahydrofolate cyclo-ligase, which participates in one-carbon metabolism. Among other uses, the product of the reaction catalyzed by this enzyme is required to produce methionine (86). Thus, it is possible that methionine levels indirectly (via tRNA^fmet^ concentrations) establish the speed of uORF translation initiation as a mechanism for translation attenuation of the main *fthC* ORF.

*Protein-binding candidates*. Candidate protein-binding RNA motifs are categorized as such because they are located immediately upstream of genes coding for nucleic acid binding proteins. These proteins commonly regulate their own expression by binding to a structured region in the 5′-UTR of their mRNA (42,87).

**Figure 4.**
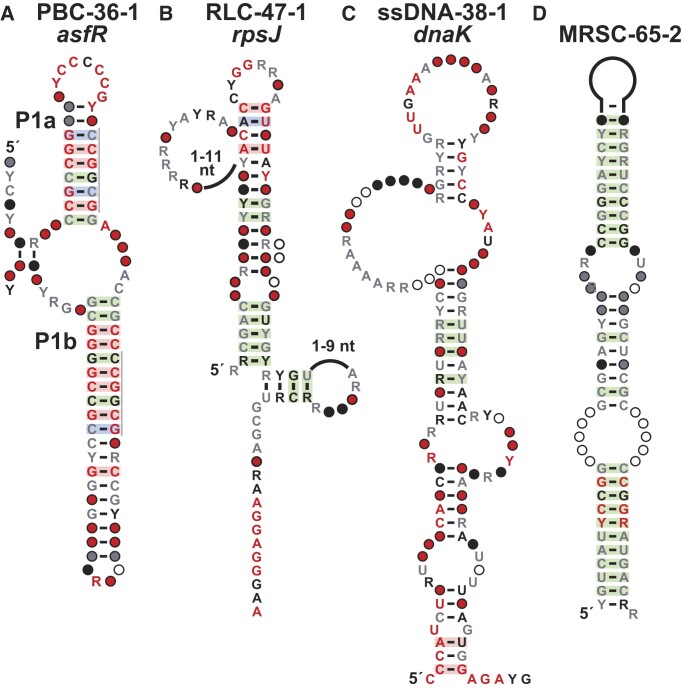
Consensus sequence and structural models of other motif types. (**A**) *asfR* motif. Repeat sequences are indicated by gray lines. (**B**) *rpsJ* motif. (**C**) *dnaK* motif. (**D**) MRSC-65–2 motif. Annotations for all motifs are as defined in Figure 1A.

The *asfR* motif (PBC-36–1). The *asfR* motif has 22 unique representatives exclusively found in the *Streptomyces* genus. It is located upstream of *asfR*, which encodes a transcription regulator that controls the expression of genes involved in the biosynthesis of clavulanic acid, a beta-lactamase inhibitor (88). The structure of the motif includes a three-stem junction (Figure 4A), where P1a and P1b have identical repeat sequences which might be recognized by the protein factor. Together with the functional annotation of the downstream gene, we include the motif in the list of protein-binding candidates.

The *rpsJ* motif (RLC-47-1). The *rpsJ* motif was originally found in *Clostridioides difficile*, and has 87 unique representatives from three genera of the Peptostreptococcaceae family (Figure 4B). It is located at the 5′-UTR of a gene encoding the small ribosomal subunit protein S10, and in *E. coli* this gene is also involved in antitermination (89). Ribosomal proteins are among the most abundant proteins in cells, and their concentrations are carefully regulated (90,91). In some examples, those protein autoregulate their expression by binding to their mRNA, leading to premature end of transcription, preventing translation of the gene, or other mechanisms that terminate the gene expression (43). Thus, motifs identified immediately upstream of genes coding for ribosomal proteins are candidates for ribosomal leader RNAs.

The *dnaK* motif (PBC-38-1). The *dnaK* motif (Figure 4C) was originally identified in the genome of *Buchnera aphidicola*. A total of 120 representatives from *Enterobacteriaceae* family, including *E. coli*, were collected. The motif is located in the IGR between the 5′-UTRs of the genes *dnaK* and *satP*, coding for a DNA replication factor (92) and acetate uptake transporter (93), respectively. The predicted structure is supported by covariation evidence and includes numerous highly conserved nucleotides distributed throughout the structure. The motif overlaps a potential promoter region, and thus could potentially be binding the *dnaK* gene product as an ssDNA.

*Mobile/repeat sequence candidates*. A motif that is found in multiple copies in a genome, especially when its orientation relative to the surrounding genes is not consistent, may be part of a transposable genetic system. Such elements are frequently associated with transposase or integrase proteins, which catalyze the movement of the genetic system in genomic DNA. An example of a mobile/repeat sequence candidate is provided below that highlights the unique features of these devices.

The MRSC-65-2 motif. With over 20 000 unique representatives from diverse phyla, the MRSC-65-2 motif is one of the most abundant motifs identified in our study. The predicted structure comprises of a single long stem (Figure 4D) that is supported by strong evidence of covariation, which is especially striking given the large number of representatives. The motif often has dozens of copies in a genome, which is more indicative of a selfish genetic element than any other type of structured nucleic acid motif. In some instances, it is located near to a gene coding for a transposase, which could potentially catalyze its distribution within genomes. The motif includes a conserved sequence repeat, UYCCGG, which could serve as a binding site for the transposase. Although the motif is depicted as a structured RNA, it is possible that this structure is relevant in its DNA form.

### High-ranking candidates

Motifs classified as high-ranking candidates (HRCs) usually are well represented by many examples, exhibit complex RNA secondary structures that are supported by covariation, and carry numerous highly conserved nucleotide positions. However, their location within genomes does not allow us to propose a functional category with reasonable confidence. HRCs are almost certain to have functions that require a complex-folded RNA structure, but additional evidence will be needed to confidently assign a biological or biochemical function. Here we provide an example of an HRC that highlights the unusual features of some of these structured RNA candidates.

**Figure 5.**
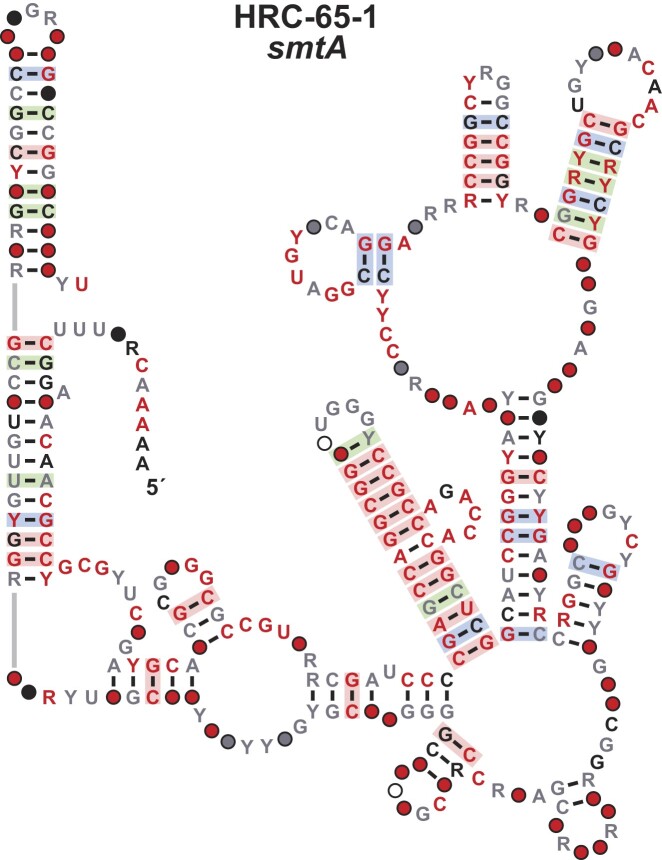
Consensus sequence and structural model the *smtA* motif. Annotations are as defined in Figure 1A. Gray lines indicate zero-length connectors.

The *smtA* motif (HRC-65-1). The *smtA* motif, or ‘Soda Lake RNA’ as we have called these representatives, is a large motif (>350 nucleotides) found exclusively in *Thioalkalivibrio* species. The motif is predicted to form a complex structure that is supported by strong evidence of covariation in multiple stems (Figure 5). *Thioalkalivibrio* is a genus of Gram-negative halophilic bacteria that include representatives found in alkaline lakes (94) (hence the name ‘Soda Lake RNA’). The motif was initially identified in the IGR adjacent to divergent genes *smtA* and *purH*.

Northern-blot assays were used to determine the orientation of the motif, proving it resides upstream of the *smtA* gene (Breaker Laboratory, unpublished findings). The *smtA* gene encodes a class I SAM-dependent methyltransferase (MTase), which uses *S*-adenosyl-L-methionine (SAM) as methyl donor (95). Class I SAM MTases represent the largest of the five classes of these enzymes (96), making it difficult to associate the motif with a specific metabolic pathway or biological process. Moreover, the motif is unusually long in comparison with most riboswitch classes (22), and therefore likely has a function different than *cis-*regulation of gene expression. Thus, we consider the *smtA* motif as high-ranking candidate (HRC) and place this among the rare examples of large ncRNAs whose functions remain to be determined.

### Concluding remarks

The results derived from implementing the GC-IGR pipeline on 50 bacterial genomes (Supplementary Figure S6 through Supplementary Figure S55) provide a detailed view of the structured ncRNA landscape in a sampling of diverse bacterial species. The current study expands the number of phyla analyzed with the GC-IGR approach from nine to 19. From this analysis, a total of 1274 candidate nucleic acid motifs have been uncovered, of which 667 candidates have been categorized into several groups of special interest (Table 1, Supplementary File S1).

Our analysis has revealed 44 novel riboswitch candidates, of which 11 are categorized as strong riboswitch candidates. These numbers are consistent with the expected pace of riboswitch discovery based on the prediction of the total number of undiscovered riboswitch classes derived from a power law distribution analysis (22,23). In addition, we consider 137 other motifs as putative *cis-*control elements, including 8 uORF candidates, 2 RNA thermometer candidates, 107 protein-binding candidates and 30 ribosome-leader sequence candidates. Thus, our findings again highlight the enormous potential for new discoveries to be made regarding the structures and functions of bacterial noncoding RNAs. Furthermore, although structured DNAs are rare, there appear to be opportunities to uncover additional DNA motifs that are relevant to transposable elements or other functions relevant to genome function and integrity.

Although the species chosen for analysis reflect a wide phylogenetic distribution, about half of the novel riboswitch candidates were discovered by searching only four genomes: *Thioalkalivibrio* (6 riboswitch candidates), *Geobacter* (5 riboswitch candidates), *Streptomyces avermitilis* (4 riboswitch candidates), and *Leptotrichia buccalis* (4 riboswitch candidates). *Thioalkalivibrio* and *Geobacter* represent organisms that thrive in extreme, isolated environments. Notably, the motifs identified in these four genomes are relatively rare and narrowly distributed. This observation might reflect the need of these species to use specialized RNA motifs to address stresses or biochemical challenges that are unique compared to those encountered by most mesophilic species. Unfortunately, the isolation of these species also might make experimental validation of the functions of these candidate RNA motifs more challenging. Unless these motifs can be tested in model bacterial species that can be more easily grown and manipulated under normal lab conditions, the technical challenges of working in the natural hosts could make their validation process more demanding.

In the present study, implementation of the GC-IGR pipeline made use of a limited version of the currently available bacterial DNA sequence database. RefSeq 80 did not include environmental DNA sequences, and therefore we supplemented our search dataset with some DNA sequence data derived from metagenomic DNA samples. Given the enormous effort required to conduct these searches, process the findings, and prepare a consensus model for each motif (14), we limited the scope of the initial DNA sequence datasets due to practical reasons.

The main bottleneck of the GC-IGR pipeline is the manual curation of multiple sequence alignments (MSAs) derived for each candidate motif. High-quality MSAs allow us to recognize structural features of the motif, identify consensus regions, and define the exact boundaries of the evolutionarily conserved structure. Manual curation is especially needed in the case of motifs that have many diverse representatives, wherein computer-generated alignments are more error-prone and tend to include large unaligned gaps (97). Future improvements in the GC-IGR pipeline could be made in optimizing computational alignment methods, which would allow each genome to be analyzed more quickly and with greater accuracy. Given the large effort needed to execute the GC-IGR pipeline at the scale described in this study, searching even larger genomic datasets is prohibitive. Thus, there is a need to develop even more efficient computational search methods that make it practical to comprehensively search all sequenced bacterial genomes. This challenge seems well suited to machine learning approaches, but this will require many structured ncRNA examples to serve as a training set. The current study provides additional examples of ncRNA candidates that helps expand the collection of possible training data to enable the development of more automated search methods.

Searching updated DNA sequence databases that include more environmental DNA sequences, can improve the quality of the structure predictions and the associations of motifs with nearby genes. This information would provide additional clues relevant to the predicted functions of the identified motifs, however, we expect most of our category assignments would remain unchanged. Rather, this additional information would most likely affect our ability to assign predicted functional categories for the unnamed motifs, for which more data may allow us to better predict their function. Although our predictions of motif functions should be considered highly tentative, we expect that the broad trends are a reasonable reflection of the enormous diversity of structured ncRNA motifs that bacteria use to contribute to their cellular functions under diverse environmental conditions.

## Supplementary Material

gkae248_Supplemental_Files

## Data Availability

The data underlying this article are available in the article and in its online supplementary material. All tables, alignment files, and structural diagrams, are available as Supplementary Files.
