# Supplementary material for: The discovery of novel noncoding RNAs in 50 bacterial genomes
Source: Nucleic Acids Res. 2024 Apr 22;52(9):5152–65. doi: 10.1093/nar/gkae248 (PMC11109978; doi:10.1093/nar/gkae248)
Supplement: gkae248_Supplemental_Files [file gkae248_supplemental_files.zip › 50 Genomes Supplementary Information Revision 1.docx]

***Supplementary Information***

**The discovery of novel noncoding RNAs in 50 bacterial genomes**

**Aya Narunsky^1^, Gadareth A. Higgs^1^, Blake M. Torres^1^, Diane Yu^1^, Gabriel Belem De Andrade^1^, Kumari Kavita^1^, and Ronald R. Breaker^1,2,3,*^**

^1^Department of Molecular, Cellular and Developmental Biology, Yale University, New Haven, CT 06511, USA; ^2^Department of Molecular Biophysics and Biochemistry, Yale University, New Haven, CT 06511, USA; ^3^Howard Hughes Medical Institute, Yale University, New Haven, CT 06511, USA

^*^To whom correspondence should be addressed. Tel: +1 203 432 9389; Email: [ronald.breaker@yale.edu](mailto:ronald.breaker@yale.edu)­­­­­

**SUPPLEMENTAL RESULTS AND DISCUSSION**

Additional descriptions of RNA motifs and additional graphics associated with the study are presented below.

**Weak Riboswitch Candidates**

The *plzA* motif (WRC-33-1). The *plzA* motif has 28 unique representatives mostly restricted to the *Borrelia* genus (**Figure S3A**). The protein product of the associated *plzA* gene is a bifunctional c-di-GMP biosensor, and previous study shows that it facilitates tick and mammalian host adaptation in *Borrelia burgdorferi* (1).

The *nrfA* motif (WRC-35-1). The *nrfA* motif has 22 unique representatives from three genera of *Pasteurellaceae* (**Figure S3B**). The protein product of the associated *nrfA* gene codes for a cytochrome c nitrite reductase, which catalyzes the six-electron reduction of nitrite to ammonia, a key step in anaerobic metabolism (2).

The *frdA* motif (WRC-35-2). The *frdA* motif has 80 unique representatives from various *Pasteurellaceae* genera (**Figure S3C**) and is located about 50 nucleotides upstream of *frdABCD* operon. This operon codes for fumarate reductase, and previous studies demonstrate that the expression of this operon in *E. coli* is affected by the concentration of alternate terminal electron acceptors oxygen, nitrate and fumarate (3).

The *ygiN* motif (WRC-36-1). The *ygiN* motif has 12 unique representatives in the *Streptomyces* genus (**Figure S3D**). It is located upstream of a gene encoding a quinol monooxygenase, which is involved in formicapyridine synthesis (4).

The *potA* motif (WRC-42-1). The *potA* motif has 15 unique representatives from *Mesoplasma* and *Mycoplasma* species (**Figure S3E**). It is located upstream of the *potA* gene, which encodes a spermidine/putrescine ABC transporter ATP-binding protein (5).

The *nupC* motif (WRC-45-1). There are only six unique representatives of the *NupC* motif, which have been identified exclusively in strains of *Oenococcus oeni* (**Figure S3F**). The associated *nupC* gene encodes for a nucleoside permease, a proton-dependent nucleoside transporter which transports adenine and pyrimidines, but not guanine (6). Under stress conditions, it is regulated by cAMP to mediate the transportation of ADP-glucose (7).

The *glpF* motif (WRC-45-2). The *glpF* motif (**Figure S3G**) is located at the 5′-UTR of the first gene in an operon coding for the glycerol uptake facilitator protein, which is involved in the uptake of glycerol and likely other carbohydrates across the membrane (8). The motif has 41 unique representatives found in *Lactobacillus* and *Oenococcus* genera.

The *mhpC* motif (WRC-47-1). There are 17 unique representatives of the *mhpC* motif found exclusively in *Clostridioides difficile* strains (**Figure S3H**). The motif is located upstream of a gene encoding a protein in the alpha/beta-hydrolase superfamily (9).

The *moaD* motif (WRC-55-1). The *moaD* motif is found upstream of a gene relevant to the biosynthesis or transport of molybdenum cofactor (Moco) (10). The motif has 11 unique examples (**Figure S3I**), of which two are found in *Hydrogenobaculum* strains and the rest were identified in environmental DNA sequence datasets. Most *Hydrogenobaculum* genomes have been isolated from extreme condition environments of Yellowstone National Park (11).

The *nifB* motif (WRC-61-2). The *nifB* motif is found upstream of a gene encoding for a nitrogenase cofactor biosynthesis protein (12), which belongs to a radical SAM protein family. There are only four examples of the motif, all identified in *Geobacter* species. The predicted structure of the motif (**Figure S3J**) includes a P3 stem-loop that includes GNRA tetraloop (13), supporting the hypothesis that this sequence indeed represents a noncoding RNA.

The *lldP* motif (WRC-61-3). The *lldP* motif has only five unique representatives, exclusively from the *Geobacter* genus (**Figure S3K**). The motif is located in the 5′-UTR of a gene coding for a lactate permease protein. In *E. coli*, this gene facilitates the uptake of L-lactate, D-lactate, and glycolate (14).

The *mrcA* motif (WRC-61-4). The *mrcA* motif has only three unique representatives in the *Geobacter* genus (**Figure S3L**). The *mrcA* gene product is penicillin binding protein 1A, which is involved in cell-wall biosynthesis (15). Due to the compelling downstream gene and its involvement in antibiotic resistance we include it in the list of riboswitch candidate.

The *mgtE*-II motif (WRC-63-2). The *mgtE*-II motif is the second motif we identify associated with the *mgtE* gene, which is annotated as coding for a magnesium transporter. It was originally identified in the *Leptotrichia* genus, and the alignment includes 63 unique sequences (**Figure S3M**), some of which are found in environmental DNA sequence datasets.

The *cbiK* motif (WRC-63-3). The *cbiK* motif has 31 unique representatives in the *Leptotrichia* genus, as well as in environmental DNA sequence datasets (**Figure S4A**). The *cbiK* gene codes for cobalt chelatase, which is in part relevant for the formation of adenosylcobalamin (coenzyme B_12_) biosynthesis (16). Riboswitches sensing adenosylcobalamin were previously described (17) and are widespread in bacteria, but their structure is very different than this newly identified motif.

The *tauB* motif (WRC-65-1). The *tauB* motif associates with a gene coding for the ATPase of an ABC-type nitrate, sulfonate of bicarbonate transporter (18). It has 27 unique representatives, exclusively from the *Thioalkalivibrio* genus (**Figure S4B**).

The *metK* motif (WRC-65-3). The *metK* motif is found exclusively in the *Thioalkalivibrio* genus and has 53 unique representatives (**Figure S4C**). It is located immediately upstream of a gene coding for methionine adenosyltransferase, which is an enzyme that uses ATP and methionine to create *S*-adenosylmethionine (SAM) (19). Many classes of SAM riboswitches have been previously reported (20). However, genetic data (Breaker Laboratory, unpublished data) suggests that this motif acts as an ON switch and enhances the expression of the downstream ORF. Because SAM is the product of the enzyme, we conclude that it is probably not the ligand sensed by the RNA.

The *sul1*-II motif (WRC-65-4). The *sul1*-II motif has 30 unique representatives in the *Thioalkalivibrio* genus (**Figure S4D**). It is located upstream of a gene encoding for a sulfate transporter (21), making it an appealing candidate for a sulfate-sensing RNA motif. A previous study identified another motif upstream of the *sul1* gene in Alphaproteobacteria, and the two motifs might represent different riboswitch classes that sense the same ligand (22).

The *mgtE*-III motif (WRC-67-1). This motif is the third example in our dataset for a motif upstream of the *mgtE* gene, which is annotated as coding for a magnesium transporter. Importantly, metal transporters are sometimes misannotated, and thus the protein may be involved in the transport of metal ions other than Mg^2+^. The *mgtE*-III motif has only six unique representatives found in *Thermoanaerobacter* genus (**Figure S4E**).

The *SCL6A* motif (WRC-70-1). The a total of 32 unique representatives of the *SLC6A* motif (**Figure S4F**) have been found in the 5′-UTR of a gene encoding the solute-binding domain of solute carrier 6 protein (SLC6). SLC6 is a representative of a large family of transporters that use sodium to facilitate the uptake of various sugars, metals, and amino acids into the cell (23, 24). The motif was identified in five species of the *Vibrio* genus and in several environmental DNA sequence datasets.

The *sdhB* motif (WRC-75-2). The *sdhB* motif has 56 unique representatives from various *Corynebacterium* species and environmental DNA sequence datasets (**Figure S4G**). It is associated with the *sdh*B gene, which codes for subunit B of succinate dehydrogenase. This complex contributes to two essential cellular processes, the citric acid cycle and the aerobic respiratory chain (25).

The *nagE* motif (WRC-80-1). The *nagE* motif was originally identified in *Clostridium botulinum*, and a total of 25 representatives have been identified in three *Clostridium* species (**Figure S4H**). The motif is associated with a gene coding for a phosphoenolpyruvate-dependent sugar phosphotransferase system. The gene product is responsible for the transport of *N*-acetylglucosamine (GlcNAc), which could be used for cell wall construction or degraded as a carbon and energy source (26, 27).

The *wrbA* motif (WRC-80-2). The *wrbA* motif is located 10-25 nucleotides upstream of the start codon of a gene that is relevant to oxidative stress conditions (28, 29). The motif is found in two *Clostridium* species, and has 45 unique representatives (**Figure S4I**).

The *fbpA* motif (WRC-80-3). The *fbpA* motif has 27 unique representatives and is found exclusively in *Clostridium* species (**Figure S4J**). It is usually located in the 5′-UTR of a gene encoding the periplasmic iron binding protein of the ferric iron transporter, and it is the first gene in the operon encoding this system (30). In several species it is found in-front of *glnQ*, which encodes a glutamine transport ATP-binding protein (31).

The *pcbC* motif (WRC-81-1). The *pcbC* motif has 28 unique representatives from three *Campylobacter* species (**Figure S4K**). The associated downstream gene codes for an enzyme that forms the beta-lactam ring of penicillin, as part of the biosynthesis of the antibiotic (32).

**Other motifs identified in the study**

The *pyrG* motif (OCRC-39-1). The *pyrG* motif was originally found in *S. agalactiae*, and includes 231 unique representatives from the *Bacili* class. It is located in the IGR between *rpoE*, whose gene product codes for subunit delta of DNA-directed RNA polymerase, and *pyrG*, encoding a CTP-synthase. In *B. subtilis*, the expression of the gene is regulated by reiterative transcription, which in the abundance of CTP stabilizes a terminator stem and prevents transcription of the downstream ORF. This leader sequence includes three elements: the sequence GGGC at the beginning of the motif, and the complementary sequences GCUCCC and GGGAGC, which base pair to form the terminator stem. All three elements are represented and highly conserved in the *pyrG* motif (**Figure S5**).

**Table S1**. Phylogenetic distribution of the genomes included in the analysis.

| Species | Accession | Phyla | Class |
| --- | --- | --- | --- |
| *Acidaminococcus intestini* RyC-MR95 | NC_016077.1 | Firmicutes | Negativicutes |
| *Agrobacterium vitis* S4 | NC_011988.1 | Proteobacteria | Alphaproteobacteria |
| *Akkermansia muciniphila* | NC_010655.1 | Verrucomicrobia | Verrucomicrobiae |
| *Arcobacter butzleri* RM4018 | NC_009850.1 | Proteobacteria | Epsilonproteobacteria |
| *Bartonella bacilliformis* KC583 | NC_008783.1 | Proteobacteria | Alphaproteobacteria |
| *Borrelia burgdorferi* B31 | NC_001318.1 | Spirochaetes | Spirochaetia |
| *Buchnera aphidicola* | NC_004061.1 | Proteobacteria | Gammaproteobacteria |
| *Campylobacter coli* CVM N29710 | NC_022347.1 | Proteobacteria | Epsilonproteobacteria |
| *Campylobacter jejuni* 81116 | NC_009839.1 | Proteobacteria | Epsilonproteobacteria |
| *Candidatus* Vesicomyosocius okutanii HA | NC_009465.1 | Proteobacteria | Gammaproteobacteria |
| *Chlamydophila caviae* GPIC | NC_003361.3 | Chlamydiae | Chlamydiia |
| *Citrobacter koseri* ATCC BAA-895 | NC_009792.1 | Proteobacteria | Gammaproteobacteria |
| *Clostridium* sp. BNL1100 | NC_016791.1 | Firmicutes | Clostridia |
| *Clostridium sticklandii* | NC_014614.1 | Firmicutes | Clostridia |
| *Clostridium tetani* E88 | NC_004557.1 | Firmicutes | Clostridia |
| *Corynebacterium pseudotuberculosis* 258 | NC_017945.2 | Actinobacteria | Actinobacteria |
| *Dehalococcoides mccartyi* 195 | NC_002936.3 | Chloroflexi | Dehalococcoidia |
| *Deinococcus geothermalis* DSM 11300 | NC_008025.1 | Deinococcus-Thermus | Deinococci |
| *Desulfobacula toluolica* Tol2 | NC_018645.1 | Proteobacteria | Deltaproteobacteria |
| *Dichelobacter nodosus* VCS1703A | NC_009446.1 | Proteobacteria | Gammaproteobacteria |
| *Dictyoglomus turgidum* DSM 6724 | NC_011661.1 | Dictyoglomi | Dictyoglomia |
| *Ehrlichia canis* str. Jake | NC_007354.1 | Proteobacteria | Alphaproteobacteria |
| *Erysipelothrix rhusiopathiae* | NC_015601.1 | Firmicutes | Erysipelotrichia |
| *Eubacterium rectale* | NC_012781.1 | Firmicutes | Clostridia |
| *Faecalitalea cylindroides* T2-87 | NC_021019.1 | Firmicutes | Erysipelotrichia |
| *Flexistipes sinusarabici* DSM 4947 | NC_015672.1 | Deferribacteres | Deferribacteres |
| *Gemmatimonas aurantiaca* T-27 | NC_012489.1 | Gemmatimonadetes | Gemmatimonadetes |
| *Geobacter* sp. M21 | NC_012918.1 | Proteobacteria | Deltaproteobacteria |
| *Haemophilus ducreyi* 35000HP | NC_002940.2 | Proteobacteria | Gammaproteobacteria |
| *Halobacteriovorax marinus* SJ | NC_016620.1 | Proteobacteria | Oligoflexia |
| *Helicobacter pylori* 26695 | NC_000915.1 | Proteobacteria | Epsilonproteobacteria |
| *Hydrogenobaculum* sp. Y04AAS1 | NC_011126.1 | Aquificae | Aquificae |
| *Kangiella koreensis* DSM | NC_013166.1 | Proteobacteria | Gammaproteobacteria |
| *Lawsonia intracellularis* N343 | NC_020127.1 | Proteobacteria | Deltaproteobacteria |
| *Leptotrichia buccalis* C-1013-b | NC_013192.1 | Fusobacteria | Fusobacteriia |
| *Listeria monocytogenes* J1816 | NC_021829.2 | Firmicutes | Bacilli |
| *Mesoplasma florum* L1 | NC_006055.1 | Tenericutes | Mollicutes |
| *Mycobacterium abscessus* | NC_018150.2 | Actinobacteria | Actinobacteria |
| *Nautilia profundicola* AmH | NC_012115.1 | Proteobacteria | Epsilonproteobacteria |
| *Oenococcus oeni* PSU-1 | NC_008528.1 | Firmicutes | Bacilli |
| *Peptoclostridium difficile* 630 | NC_009089.1 | Firmicutes | Clostridia |
| *Prevotella ruminicola* 23 | NC_014033.1 | Bacteroidetes | Bacteroidia |
| *Prosthecochloris aestuarii* DSM 271 | NC_011059.1 | Chlorobi | Chlorobia |
| *Streptobacillus moniliformis* DSM 12112 | NC_013515.1 | Fusobacteria | Fusobacteriia |
| *Streptococcus agalactiae* 2603VR | NC_004116.1 | Firmicutes | Bacilli |
| *Streptomyces avermitilis* MA-4680 | NC_003155.5 | Actinobacteria | Actinobacteria |
| *Thermoanaerobacter* sp. X513 | NC_014538.1 | Firmicutes | Clostridia |
| *Thioalkalivibrio* sp. K90mix | NC_013889.1 | Proteobacteria | Gammaproteobacteria |
| *Vibrio anguillarum* 775 | NC_015633.1 | Proteobacteria | Gammaproteobacteria |
| *Wolinella succinogenes* DSM 1740 | NC_005090.1 | Proteobacteria | Epsilonproteobacteria |
| TOTAL |  | 16 | 23 |


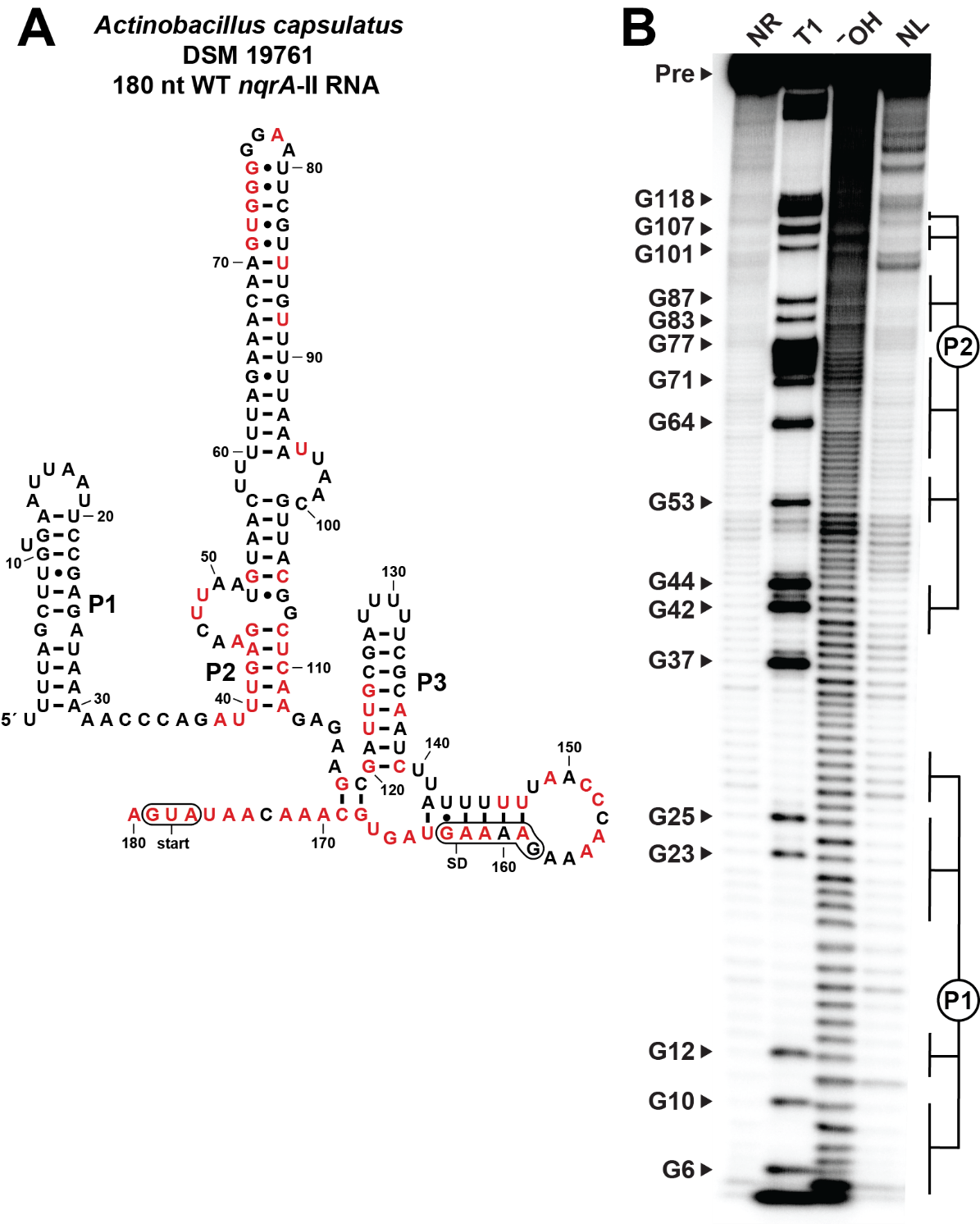


**Figure S1.** In-line probing analysis of a representative *nqrA*-II motif RNA. (**A**) Sequence and structural model of a representative *nqrA*-II RNA motif from *Actinobacillus capsulatus* DSM 19761. Red nucleotides identify highly conserved positions in the *nqrA*-II motif consensus (**Figure 1A**). (**B**) In-line probing analysis of the *nqrA*-II motif from *A. capsulatus* conforms to the predicted consensus model structure. The image depicts an autoradiogram of the products of in-line probing reactions with trace amounts of 5´ ^32^P-labeled RNA separated by using denaturing (8 M urea) 10 % polyacrylamide gel electrophoresis (PAGE). NR indicates no reaction, T1 indicates partial digestion of RNA using RNase T1 (cleaves after G nucleotides), ^‒^OH indicates partial digestion of RNA using alkaline conditions (cleaves after every nucleotide), NL indicates no ligand was added to the in-line probing reaction.

Methods: In-line probing assays were conducted as described elsewhere (see main text) The RNAs were prepared by transcription of a double-stranded PCR product made by amplification of the following synthetic DNAs:

nqrA-II-180F 5′-

TAATACGACTCACTATAggTTTTAGCTTGTGAATTAATTCCGAGATAAAAACCCAGATTTGAGAACTTAATGTAACTTTTTAGAAACAAGTGGGGGAATTCGTTTGTTTTTAAATTA

nqrA-II-180R 5′-

TCATATTGTTTGCACTACTTTTCTTTTGGGTTAAAAAATAAGATTGCGAAAAATCGCAATCGCTTCTCTTGAGCCGTAACGTTAATTTAAAAACAAACGA


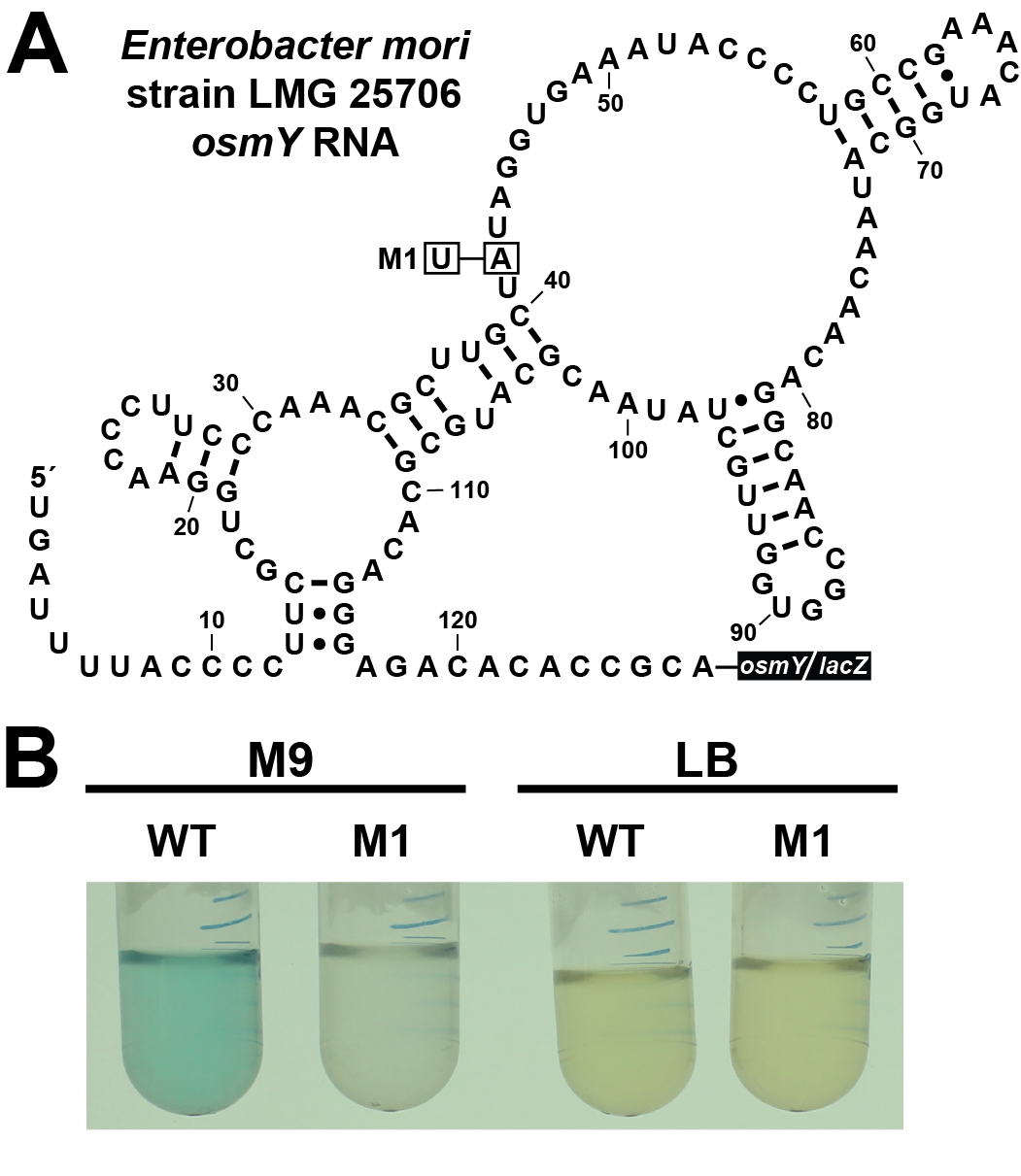


**Figure S2.** Evidence for riboswitch function by a representative *opuB* motif RNA. (**A**) Sequence and secondary structure model for the *opuB* motif representative from *Enterobacter mori* strain LMG 25706. Boxed letters designate the A42U mutation in construct M1. The construct, derived from the *osmY* (formerly annotated as *opuB*) gene was fused in-frame with the *lacZ* reporter gene. (**B**) β-galactosidase reporter assays used to assess the level of gene expression of WT and mutant (M1) *opuB* motif RNAs in cells cultured with minimal (M9) or rich (LB) media. Blue color indicates high reporter gene expression.

Methods: A translational reporter construct was made by fusing DNA corresponding to the *opuB* motif (**Figure 1F**), including the first eight codons of the downstream *osmY* gene to the *lacZ* coding region of *E. coli* in a pRS414 plasmid under the control of a *thiC* promoter. The WT and M1 (A42U) constructs were transformed into *E. coli* BW 25113 cells. Reporter assays were performed in liquid culture, by diluting overnight cultures of *E. coli* carrying either the WT or the M1 riboswitch-reporter fusion constructs in M9 minimal media (1/100) or lysogeny broth (LB) (1/10) supplemented with carbenicillin (100 µg mL^-1^) and X-gal (5-bromo-4-chloro-3-indolyl-β-D-galactopyranoside) at 100 µg mL^-1^. The cultures were incubated at 37°C for development of blue color (indicative of high β-galactosidase activity) and images were recorded. The experiments were conducted in triplicate, with representative photographs depicted.

**Figure S3**. Consensus sequence and structural models of several weak riboswitch candidates. (**A**) *plzA* motif. (**B**) *nrfA* motif. (**C**) *frdA* motif. (**D**) *ygiA* motif. (**E**) *potA* motif. (**F**) *nupC* motif. (**G**) *glpF* motif. (**H**) *mhpC* motif. (**I**) *moaD* motif. (**J**) *nifB* motif. (**K**) *lldP* motif. (**L**) *mrcA* motif. (**M**) *MgtE*-II motif. Annotations for all motifs are as defined in **Figure 1A**.

**Figure S4**. Consensus sequence and structural models of several additional weak riboswitch candidates. (**A**) *cbiK* motif. (**B**) *tauB* motif. (**C**) *metK* motif. (**D**) *sul1*-II motif. (**E**) *mgtE*-III motif. (**F**) *SLC6A* motif. (**G**) *sdhB* motif. (**H**) *nagE* motif. (**I**) *wrbA* motif. (**J**) *fbpA* motif. (**K**) *pcbC* motif. Annotations for all motifs are as defined in **Figure 1A**.

**Figure S5.** Consensus sequence and structural models of the *pyrG* motif. Annotations are as defined in **Figure 1A**.

**IGR Plots of the Genomes Analyzed in the Study.** Each plot represents the % G and C nucleotide content versus the length of all intergenic regions (IGRs) present in an organism. The annotations defining each point type are presented in the key for each plot. Note that most IGRs containing a known noncoding RNA (colored symbols) tend to reside in the upper right portion of the cloud of IGRs, reflecting the fact that IGRs serving as templates for the production of structured noncoding RNAs tend to be more GC-rich and longer than the typical IGR in most species. This trend is different for bacterial species that have GC-rich genomes, such as *Streptomyces avermitilis*. The clustering of IGRs relevant to noncoding RNAs reveals the likely locations of IGRs that carry undiscovered noncoding RNA classes.

**Fig S6**. The IGRs from the *H. pylori* genome sorted by IGR length and GC content.

**Fig S7**. The IGRs from the *B. burgdorferi* genome sorted by IGR length and GC content.

**Fig S8**. The IGRs from the *D. mccartyi* genome sorted by IGR length and GC content.

**Fig S9**. The IGRs from the *H. ducreyi* genome sorted by IGR length and GC content.

**Fig S10.** The IGRs from the *S. avermitilis* genome sorted by IGR length and GC content.

**Fig S11.** The IGRs from the *C. caviae* genome sorted by IGR length and GC content.

**Fig S12.** The IGRs from the *B. aphidicola* genome sorted by IGR length and GC content.

**Fig S13.** The IGRs from the *S. agalactiae* genome sorted by IGR length and GC content.

**Fig S14.** The IGRs from the *C. tetani* genome sorted by IGR length and GC content.

**Fig S15.** The IGRs from the *W. succinogenes* genome sorted by IGR length and GC content.

**Fig S16.** The IGRs from the *M. florum* genome sorted by IGR length and GC content.

**Fig S17.** The IGRs from the *E. canis* genome sorted by IGR length and GC content.

**Fig S18.** The IGRs from the *D. geothermalis* genome sorted by IGR length and GC content.

**Fig S19.** The IGRs from the *O. oeni* genome sorted by IGR length and GC content.

**Fig S20.** The IGRs from the *B. bacilliformis* genome sorted by IGR length and GC content.

**Fig S21.** The IGRs from the *P. difficile* genome sorted by IGR length and GC content.

**Fig S22.** The IGRs from the *D. nodosus* genome sorted by IGR length and GC content.

**Fig S23.** The IGRs from the *Candidatus V. okutanii* genome sorted by IGR length and GC content.

**Fig S24.** The IGRs from the *C. koseri* genome sorted by IGR length and GC content.

**Fig S25.** The IGRs from the *C. jejuni* genome sorted by IGR length and GC content.

**Fig S26.** The IGRs from the *A. butzleri* genome sorted by IGR length and GC content.

**Fig S27.** The IGRs from the *A. muciniphila* genome sorted by IGR length and GC content.

**Fig S28.** The IGRs from the *P. aestuarii* genome sorted by IGR length and GC content.

**Fig S29.** The IGRs from the *Hydrogenobaculum sp.* genome sorted by IGR length and GC content.

**Fig S30.** The IGRs from the *D. turgidum* genome sorted by IGR length and GC content.

**Fig S31.** The IGRs from the *A. vitis* genome sorted by IGR length and GC content.

**Fig S32.** The IGRs from the *N. profundicola* genome sorted by IGR length and GC content.

**Fig S33.** The IGRs from the *G. aurantiaca* genome sorted by IGR length and GC content.

**Fig S34.** The IGRs from the *E. rectale* genome sorted by IGR length and GC content.

**Fig S35.** The IGRs from the *Geobacter sp.* genome sorted by IGR length and GC content.

**Fig S36.** The IGRs from the *K. koreensis* genome sorted by IGR length and GC content.

**Fig S37.** The IGRs from the *L. buccalis* genome sorted by IGR length and GC content.

**Fig S38.** The IGRs from the *S. moniliformis* genome sorted by IGR length and GC content.

**Fig S39.** The IGRs from the *Thioalkalivibrio sp.* genome sorted by IGR length and GC content.

**Fig S40.** The IGRs from the *P. ruminicola* genome sorted by IGR length and GC content.

**Fig S41.** The IGRs from the *Thermoanaerobacter sp.* genome sorted by IGR length and GC content.

**Fig S42.** The IGRs from the *C. sticklandii* genome sorted by IGR length and GC content.

**Fig S43.** The IGRs from the *E. rhusiopathiae* genome sorted by IGR length and GC content.

**Fig S44.** The IGRs from the *V. anguillarum* genome sorted by IGR length and GC content.

**Fig S45.** The IGRs from the *F. sinusarabici* genome sorted by IGR length and GC content.

**Fig S46.** The IGRs from the *A. intestini* genome sorted by IGR length and GC content.

**Fig S47.** The IGRs from the *H. marinus* genome sorted by IGR length and GC content.

**Fig S48.** The IGRs from the *Clostridium sp.* genome sorted by IGR length and GC content.

**Fig S49.** The IGRs from the *C. pseudotuberculosis* genome sorted by IGR length and GC content.

**Fig S50.** The IGRs from the *M. abscessus* genome sorted by IGR length and GC content.

**Fig S51.** The IGRs from the *D. toluolica* genome sorted by IGR length and GC content.

**Fig S52.** The IGRs from the *L. intracellularis* genome sorted by IGR length and GC content.

**Fig S53.** The IGRs from the *F. cylindroides* genome sorted by IGR length and GC content.

**Fig S54.** The IGRs from the *C. botulinum* genome sorted by IGR length and GC content.

**Fig S55.** The IGRs from the *C. coli* genome sorted by IGR length and GC content.

**References**

1. Groshong,A.M., Grassmann,A.A., Luthra,A., McLain,M.A., Provatas,A.A., Radolf,J.D. and Caimano,M.J. (2021) PlzA is a bifunctional c-di-GMP biosensor that promotes tick and mammalian host-adaptation of *Borrelia burgdorferi*. *PLoS Pathog.*, **17**.

2. Campeciño,J., Lagishetty,S., Wawrzak,Z., Alfaro,V.S., Lehnert,N., Reguera,G., Hu,J. and Hegg,E.L. (2020) Cytochrome *c* nitrite reductase from the bacterium *Geobacter lovleyi* represents a new NrfA subclass. *J. Biol. Chem.*, **295**, 11455-11465.

3. Jones,H.M. and Gunsalus,R.P. (1987) Regulation of *Escherichia coli* fumarate reductase (*Frdabcd*) operon expression by respiratory electron-acceptors and the *Fnr* gene product. *J. Bacteriol.*, **169**, 3340-3349.

4. Qin,Z.W., Devine,R., Hutchings,M.I. and Wilkinson, B.(2019) A role for antibiotic biosynthesis monooxygenase domain proteins in fidelity control during aromatic polyketide biosynthesis. *Nat. Commun.*, **10**.

5. Kashiwagi,K., Miyamoto,S., Nukui,E., Kobayashi,H. and Igarashi K. (1993) Functions of *potA* and *potD* proteins in spermidine-preferential uptake system in *Escherichia coli*. *J. Biol. Chem.*, **268**, 19358-19363.

6. Mygind,B. and Munch-Petersen, A. (1975) Transport of pyrimidine nucleosides in cells of *Escherichia Coli* K-12. *Eur. J. Biochem..*, **59**, 365-372.

7. Almagro,G., Viale,A.M., Montero,M., Muñoz,F.J., Baroja-Fernández,E., Mori,H. and Pozueta-Romero,J. (2018) A cAMP/CRP-controlled mechanism for the incorporation of extracellular ADP-glucose in *Escherichia coli* involving NupC and NupG nucleoside transporters. *Sci. Rep.*, **8**.

8. Stroud,R.M., Miercke,L.J., O'Connell,J., Khademi,S., Lee,J.K., Remis,J., Harries,W., Robles,Y. and Akhavan,D. (2003) Glycerol facilitator GlpF and the associated aquaporin family of channels. *Curr. Opin. Struct. Biol.*, **13**, 424-431.

9. Li,C., Hassler,M. and Bugg,T.D.H. (2008) Catalytic promiscuity in the alpha/beta-hydrolase superfamily: Hydroxamic acid formation, C-C bond formation, ester and thioester hydrolysis in the C-C hydrolase family. *ChemBioChem*, **9**, 71-76.

10. Williams,M.J., Kana,B.D. and Mizrahi,V. (2011) Functional analysis of molybdopterin biosynthesis in *Mycobacteria* identifies a fused molybdopterin synthase in *Mycobacterium tuberculosis*. *J. Bacteriol.*, **193**, 98-106.

11. Romano,C., D'Imperio,S., Woyke,T., Mavromatis,K., Lasken,R., Shock,E.L. and McDermott,T.R. (2013) Comparative genomic analysis of phylogenetically closely related *Hydrogenobaculum* sp isolates from Yellowstone National Park. *Appl. Environ. Microbiol.*, **79**, 2932-2943.

12. Li, Q., Zhang,H.W., Zhang,L.Q. and Chen,S.F. (2021) Functional analysis of multiple *nifB* genes of *Paenibacillus* strains in synthesis of Mo-, Fe- and V-nitrogenases. *Microb. Cell Factories*, **20**.

13. Correll,C.C. and Swinger,K. (2003) Common and distinctive features of GNRA tetraloops based on a GUAA tetraloop structure at 1.4 angstrom resolution. *RNA*, **9**, 355-363.

14. Núñez,M.F., Kwon,O., Wilson,T.H., Aguilar,J., Baldoma,L. and Lin,E.C. (2002) Transport of L-Lactate, D-Lactate, and glycolate by the LldP and GlcA membrane carriers of *Escherichia coli*. *Biochem. Biophys. Res. Commun.*, **290**, 824-829.

15. Meberg,B.M., Sailer,F.C., Nelson,D.E. and Young,K.D. (2001) Reconstruction of *Escherichia coli* *mrcA* (PBP 1a) mutants lacking multiple combinations of penicillin binding proteins. *J. Bacteriol.*, **183**, 6148-6149.

16. rindley,A.A.,Raux, E.,Leech,H.K., Schubert,H.L. and Warren,M.J. (2003) A story of chelatase evolution - Identification and characterization of a small 13-15-kda "ancestral" cobaltochelatase (CbiX(s)) in the archaea. *J. Biol. Chem.*, **278**, 22388-22395.

17. Nahvi,A., Barrick,J.E. and Breaker,R.R. (2004) Coenzyme B12 riboswitches are widespread genetic control elements in prokaryotes. *Nucleic Acids Res*, **32**, 143-150.

18. Li,W.L., Cong,Q., Pei,J.M., Kinch,L.N. and Grishin,N.V. (2012) The ABC transporters in *Candidatus* Liberibacter asiaticus. *Proteins*, **80**, 2614-2628.

19. Markham,G.D. and Pajares,M.A. (2009) Structure-function relationships in methionine adenosyltransferases. *Cell. Mol. Life Sci.*, **66**, 636-648.

20. McCown,P.J., Corbino,K.A., Stav,S., Sherlock,M.E. and Breaker,R.R. (2017) Riboswitch diversity and distribution. *RNA*, **23**, 995-1011.

21. Kankipati,H.N., Rubio-Texeira,M., Castermans,D., Diallinas,G. and Thevelein,J.M. (2015) Sul1 and Sul2 sulfate transceptors signal to protein kinase A upon exit of sulfur starvation. *J. Biol. Chem.*, **290**, 10430-10446.

22. Weinberg,Z., Lünse,C.E., Corbino,K.A., Ames,T.D., Nelson,J.W., Roth,A., Perkins,K.R., Sherlock,M.E. and Breaker,R.R. (2017) Detection of 224 candidate structured RNAs by comparative analysis of specific subsets of intergenic regions. *Nucleic Acids Res.*, **45**, 10811-10823.

23. Bröer,S. and Gether,U. (2012) The solute carrier 6 family of transporters. *Br. J. Pharmacol.* **167**, 256-278.

24. Razavi,A.M., Khelashvili,G. and Weinstein,H. (2018) How structural elements evolving from bacterial to human SLC6 transporters enabled new functional properties. *BMC Biol.*, **16**, 31.

25. Oyedotun,K.S. and Lemire,B.D. (2004) The quaternary structure of the *Saccharomyces cerevisiae* succinate dehydrogenase. Homology modeling, cofactor docking, and molecular dynamics simulation studies. *J. Biol. Chem.*, **279**, 9424-9431.

26. Lengeler,J.W., Jahreis,K. and Wehmeier,U.F. (1994) Enzymes II of the phospho enol pyruvate-dependent phosphotransferase systems: their structure and function in carbohydrate transport. *Biochim Biophys Acta Bioenerg*, **1188**, 1-28.

27. Plumbridge,J.A. (1989) Sequence of the *Nagbacd* operon in *Escherichia Coli* K12 and pattern of transcription within the *nag* regulon. *Mol. Microbiol.*, **3**, 505-515.

28. Grandori,R., Khalifah,P., Boice,J.A., Fairman,R., Giovanielli,K. and Carey,J. (1998) Biochemical characterization of WrbA, founding member of a new family of multimeric flavodoxin-like proteins. *J. Biol. Chem.*, **273**, 20960-20966.

29. Dabravolski,S.A. (2020) Evolutionary aspects of the *Viridiplantae* nitroreductases. *J Genet Eng Biotechnol*, **18**.

30. Steunou,A.S., Bourbon,M.L., Babot,M., Durand,A., Liotenberg,S., Yamaichi,Y. and Ouchane,S. (2020) Increasing the copper sensitivity of microorganisms by restricting iron supply, a strategy for bio-management practices. *Microb. Biotechnol.*, **13**, 1530-1545.

31. Masters,P.S. and Hong,J.S. (1981) Genetics of the glutamine transport system in *Escherichia coli*. *J. Bacteriol.*, **147**, 805-819.

32. Martín,J.F. and Gutiérrez,S. (1995) Genes for beta-lactam antibiotic biosynthesis. *Antonie Van Leeuwenhoek*, **67**, 181-200.

33. Meng,Q., Turnbough,C.L. Jr and Switzer,R.L. (2004) Attenuation control of *pyrG* expression in *Bacillus subtilis* is mediated by CTP-sensitive reiterative transcription. *Proc. Natl. Acad. Sci. U.S.A.*, **101**, 10943-10948.
